# Enhancement of antebrachiocarpal arthrodesis using a collagen–chitosan composite enriched with advanced platelet-rich fibrin in a rabbit model

**DOI:** 10.1038/s41598-026-41968-4

**Published:** 2026-06-17

**Authors:** Gannah- Samy, Rawan- Hagras, Awad Rizk, Emad Tolba, Zainab A. Ramadan, Ekramy Elmorsy, Gamal Karrouf

**Affiliations:** 1 Department of Surgery, Anesthesiology, and Radiology, Faculty of Veterinary Medicine, University of Mansoura, Mansoura, 35516 Egypt; 2 Polymers and Pigments Department, National Research Centre, 33 El Bohouth St, P.O. 12622, Dokki, Giza Egypt; 3 Department of Diagnostic and Interventional Radiology, Faculty of Medicine, Mansoura University, Mansoura, 35516 Egypt; 4 Center for Health Research, Northern Border University, P.O. Box 1321, Arar, 91431 Saudi Arabia

**Keywords:** Advanced platelet-rich fibrin, Arthrodesis, Collagen-Chitosan, Rabbits, Anatomy, Diseases, Health care, Medical research, Rheumatology

## Abstract

Arthrodesis is a pivotal surgical intervention for achieving joint stability and alleviating pain in cases of severe joint destruction. However, attaining reliable bone fusion remains challenging, frequently requiring adjunctive biomaterials to optimize osteogenesis. The present study was designed to investigate the combined regenerative effect of a collagen–chitosan composite integrated with A-PRF on antebrachiocarpal joint arthrodesis in a rabbit model. This experimental study involved 16 clinically healthy male New Zealand White rabbits. All animals underwent surgical curettage of the antebrachiocarpal joint cartilage. The rabbits were randomly allocated into two groups: a Control group (C) and a treatment group (COL/Cs -A-PRF). Over the 12 weeks, the Col/Cs/A-PRF group demonstrated marked radiological improvement. Radial cortical thickness (RCT) increased from 0.91 ± 0.13 mm to 1.4 ± 0.05 mm (*p* < 0.001). Radial bone mineral density (RBMD) progressively increased, reaching approximately 780 HU (*p* = 0.0007). Intra-articular tissue mineral density increased to around 370 HU. Joint space narrowing progressed from approximately 1.0 mm to 0.3 mm (*p* < 0.0001), with the fusion ratio reaching nearly 70% (*p* < 0.0001). Additionally, carpal bone density increased to approximately 990 HU (*p* < 0.0001). The presented findings demonstrate that the collagen–chitosan composite augmented with A-PRF significantly improves bone regeneration and joint fusion in a rabbit model of antebrachiocarpal arthrodesis, highlighting its innovative and translational potential.

## Introduction

Antebrachiocarpal arthrodesis is a surgical procedure used to treat severe injuries or deformities of the carpus, such as hyperextension injuries, luxation, comminuted fractures, or congenital deformities like radial agenesis. In dogs and cats, this procedure is often performed as a salvage technique when other treatments are not viable, especially for chronic instability, severe trauma, or congenital conditions^1^. Various techniques have been established over the years to achieve successful arthrodesis in both human and veterinary orthopedic applications. Traditional methods often rely on the curettage of articular cartilage and application of metallic fixation devices such as plates and screws^2^. While the procedure can lead to good functional outcomes and limb salvage, complications such as infection, implant failure, and reduced limb function are possible, and the prognosis depends on the underlying condition and the surgical technique^3^.

Tissue engineering has developed as a promising approach for bone fusion, especially in cases where traditional bone grafts are limited by supply or complications. The core strategy involves combining scaffolds often made from biomimetic materials like collagen, hydroxyapatite, ceramics, or biodegradable polymers with stem cells and growth factors to create an environment that supports new bone growth and vascularization^4,5^. Chitosan-based scaffolds are highly effective in promoting osteogenesis, These scaffolds support the attachment, proliferation, and differentiation of osteoprogenitor and mesenchymal stem cells into osteoblasts, as demonstrated by increased expression of osteogenic genes, alkaline phosphatase activity, and mineralized matrix formation both in vitro and in vivo^6^.

The osteogenic potential of chitosan scaffolds can be further enhanced by combining chitosan with other biopolymers and bioactive or metal-organic frameworks, which improve mechanical strength, porosity, and biological activity^6–8^. Additionally, loading chitosan scaffolds with bioactive molecules, such as platelet-rich fibrin or osteogenic drugs, can further stimulate bone cell growth and differentiation^9^.

Marine collagen is a key material for scaffolds in skin, bone, and cartilage regeneration. It increases bone mineral density, supports osteoblast maturation, and aids in the prevention and treatment of osteoporosis and other bone-related diseases^10^. Its excellent biocompatibility and lower zoonotic risk make it suitable for medical implants and tissue engineering constructs^10,11^.

Collagen-chitosan membranes and scaffolds exhibit enhanced tensile strength, controlled degradation rates, and high porosity, which are favorable for cell adhesion and proliferation^12,13^.

Advanced platelet-rich fibrin (A-PRF) is a specialized form of platelet concentrate derived from a patient’s blood, designed to enhance tissue regeneration and healing^14^. It is produced using a specific centrifugation protocol that yields a fibrin matrix rich in platelets, leukocytes, and growth factors, which are gradually released to support tissue repair^15^. A-PRF enhances mineralization, calcium production, and early cell differentiation in osteoblast-like cells^16^.

Multislice computed tomography (MSCT) scanners, equipped with multiple rows of detectors, have significantly advanced bone imaging by enabling rapid acquisition of high-resolution, volumetric datasets^17^. These systems allow for thin-slice imaging (as small as 0.5 mm), which provides detailed visualization of bone microarchitecture and facilitates multiplanar and three-dimensional reconstructions essential for musculoskeletal assessment and surgical planning^18,19^. It is also highly effective in evaluating bone density and quality, with Hounsfield unit (HU) measurements, correlating strongly with bone volume fraction and microstructural parameters^20^.

This study aims to evaluate the efficacy of a collagen–chitosan composite scaffold enriched with autologous advanced platelet-rich fibrin (A-PRF) in enhancing antebrachiocarpal arthrodesis in a rabbit model. The study also seeks to assess bone fusion non-invasively using multislice computed tomography (MSCT).

## Materials and methods

### Animal model description and care protocol

Sixteen clinically healthy male New Zealand White rabbits (4.0 ± 0.3 months old; 2.5 ± 0.5 kg) were used in this study. The animals were bred and obtained from the Medical Experimental Research Center (MERC), Faculty of Medicine, Mansoura University, and were acclimatized for two weeks before experimentation. Rabbits were housed under controlled environmental conditions at the Veterinary Teaching Hospital, Faculty of Veterinary Medicine, Mansoura University, Egypt, with a constant temperature of 22 ± 1 °C, 55% relative humidity, and a 12-h light–dark cycle. All animals received a standard pelleted diet and water ad libitum throughout the study. Experimental procedures were conducted in accordance with the Guide for the Care and Use of Laboratory Animals and were approved by the Institutional Animal Care and Use Committee of Mansoura University **(Ref: MUACUC (VM.MS.24.04.132)).** The study also complied with the ARRIVE guidelines.

### Study design

**Fig. 1:**
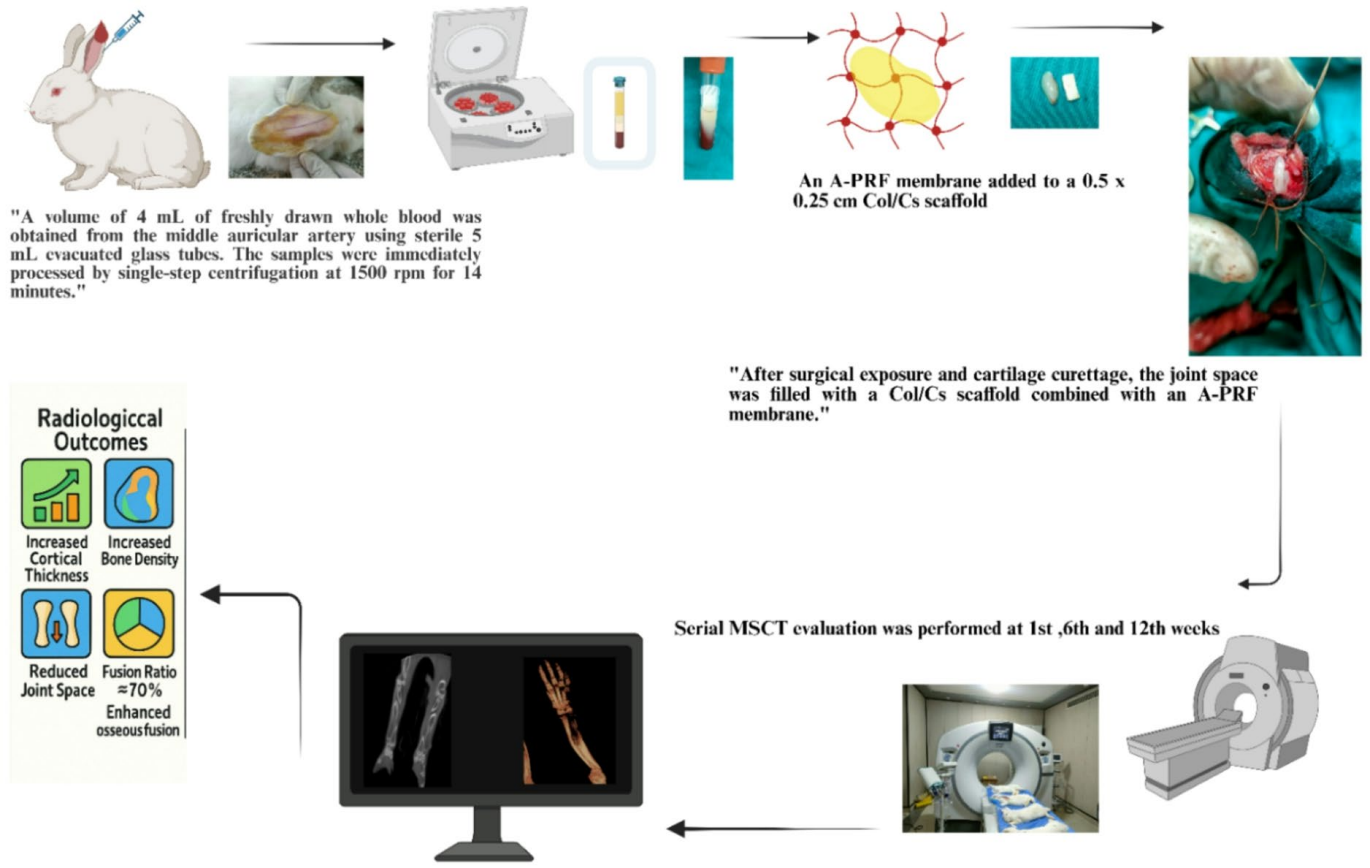
Schematic illustration of A-PRF preparation, scaffold implantation, and radiological evaluation in a rabbit antebrachiocarpal arthrodesis model.

Sixteen rabbits underwent complete surgical curettage of the articular cartilage to reach the subchondral bone. After the procedure, the animals were randomly allocated into two groups: a control group (C), no scaffold implantation, and a treatment group (Col/Cs-A-PRF), in which a collagen–chitosan composite scaffold enriched with autologous advanced platelet-rich fibrin was used (Fig. 1**).**

### Preparation and microstructure characterization of collagen/chitosan (Col/Cs) composite scaffolds

Hydrolyzed deep-sea marine collagen peptides were obtained from Beijing SEMNL Biotechnology (China). Chitosan (Cs) with a molecular weight of 1 × 10¹² g/mol was supplied by Sigma Aldrich (Germany).

To fabricate the collagen–chitosan hydrogels, a combination of freeze-gelation and freeze-drying techniques was employed. In brief, 2 g of chitosan was dissolved in 50 mL of 1% (v/v) acetic acid solution under continuous stirring for 1 h at room temperature. Concurrently, 2 g of marine collagen was dissolved in 50 mL of distilled water at 37 °C for 10 min. Equal volumes of both solutions were then combined and gently stirred for an additional hour to ensure homogeneity. The resulting Col/Cs mixture was transferred into sterile Petri dishes (90 mm diameter × 15 mm depth), covered with lids, and frozen at − 20 °C for 24 h.

Following freezing, the samples were immersed in a NaOH/ethanol solution and kept at − 20 °C overnight. Subsequently, the formed hydrogels were carefully removed from the dishes and rinsed thoroughly with ethanol to eliminate any residual NaOH. The hydrogels were then refrozen at − 20 °C for another 24 h before undergoing lyophilization at − 56 °C to remove the frozen solvent completely.

For structural analysis, X-ray diffraction (XRD) was performed using a Bruker D8 Advance X-ray diffractometer (Germany), operating at 40 kV voltage and 40 mA current, with Cu-Kα radiation (λ = 1.5406 Å). Diffraction patterns were recorded over a 2θ range of 5° to 80° to assess the crystalline structure and phase composition of the scaffold material.

### Preparation of autologous advanced platelet-rich fibrin (A-PRF)

Blood samples of 4 mL were collected directly into sterile evacuated glass tubes (Shanghai Goldenwell Medical Technology Co., Ltd., China) and, within 60–90 Sect^21^., centrifuged at 1500 rpm for 14 min^22^. The A-PRF clot is then gently compressed between sterile gauze and applied immediately into the joint space.

### Operative procedure

A preoperative antibiotic, Cefotaxime sodium, was administered intravenously at a dosage of 50 mg/kg (Cefotax, EIPICO Pharmaceutical Company, Egypt). General anesthesia was induced with intramuscular injection of 7.5 mg/kg xylazine (Xylaject, 20 mg/ml; Adwia Co., Egypt) and 40 mg/kg ketamine hydrochloride (Ketamine 50 mg/10 ml, Rotexmedica, Germany). Under complete aseptic conditions and in lateral recumbency, a 3 cm longitudinal incision was made over the dorsal aspect of the right carpus, starting from the lower third of the radius and extending toward the carpometacarpal joint. And the procedure was continued as mentioned by^23^. In the control group, no scaffold was used, whereas in the Col/Cs-A-PRF group, a 0.5 × 0.25 cm scaffold was placed in the joint space, along with the A-PRF membrane.

### Postoperative care and follow-up

Cefotaxime sodium was continued for 5 days, along with meloxicam (Anticox II 15 mg, ADWIA, Egypt), administered at a dose of 0.6 mg/kg once daily for 3 days. One week after surgery, the wound was dressed with povidone-iodine and treated with a bivatracin antibiotic spray (ECAP, Egypt), then the fiberglass cast was reapplied. The rabbits were monitored daily for activity and movement until the end of the study. The rabbits were euthanized using an intravenous injection of thiopental sodium. (Dose of 100–150 mg/kg) as a rapid bolus.

### Computed tomography imaging and analysis

Quantitative and qualitative CT-based criteria were evaluated to assess the progression of ankylosis over a 90-day postoperative period in vivo. Multislice computed tomography scans were acquired on postoperative days 7, 45, and 90 using a GE Healthcare 128-slice CT scanner (Revolution EVO, Waukesha, WI, USA). Imaging protocols were configured with the following parameters: 100–120 kVp, 150–250 mAs (automatically adjusted via exposure modulation), window width of 25–50 cm, large body field of view, slice thickness of 0.625 mm, reconstruction interval of 0.625 mm, and a pitch of approximately 0.984:1 at a table speed of ~ 40 mm/s (helical acquisition mode). Images were reconstructed using both bone and soft tissue algorithm kernels to optimize the visualization of relevant anatomical structures.

### Tomographic image assessment

All radiographic assessments were conducted by a single, experienced radiologist with over ten years of specialized practice, ensuring standardized interpretation and minimizing observer-related discrepancies. Measurements were systematically obtained from multiple image slices, and mean values were calculated to enhance accuracy in the evaluation of joint fusion progression.

Radial cortical thickness was assessed in millimeters (mm) at specific, predetermined points located in the middle of the distal end using a digital caliper on the CT workstation.

Radial bone mineral density (RBMD), expressed in Hounsfield units (HU), was evaluated at the distal radius. The region of interest (ROI), where RBMD was measured, was consistently set to approximately 5 mm² and positioned at the interface between the cortical bone and the underlying medulla. Similar measurement techniques were applied to assess bone mineral density in the ulna and carpal bones, as well as in both the radial and ulnar bones.

Within the intra-articular region, the site of the region of interest (ROI) changed with the healing phase: on postoperative Day 7, the ROI was placed within the joint space to evaluate intra-articular changes, whereas in later stages of healing, it was positioned within the residual joint space.

The fusion ratio was determined according to the following formula:

**Fusion ratio = (Sum of widths of fused segments/Sum of widths of the joint surface) × 100**, and the average value was then calculated.

The fusion grades were adapted from the classification system proposed by **Mehlhorn et al. (2020).** Failed fusion is considered when it is less than 33%, while partial fusion occurs when it is between 34% and 66%, and when it is 67% or more, complete fusion is considered^24^.

### Statistical analysis

All statistical analyses were conducted using GraphPad Prism software, version 8.3.4 (GraphPad Software, San Diego, CA, USA) https://www.graphpad.com/. Data are expressed as mean ± SD. Normality was tested using the Shapiro–Wilk test. Group and time effects were assessed using a two-way ANOVA with repeated-measures. A p-value < 0.05 was considered statistically significant.

## Results

**Fig. 2:**
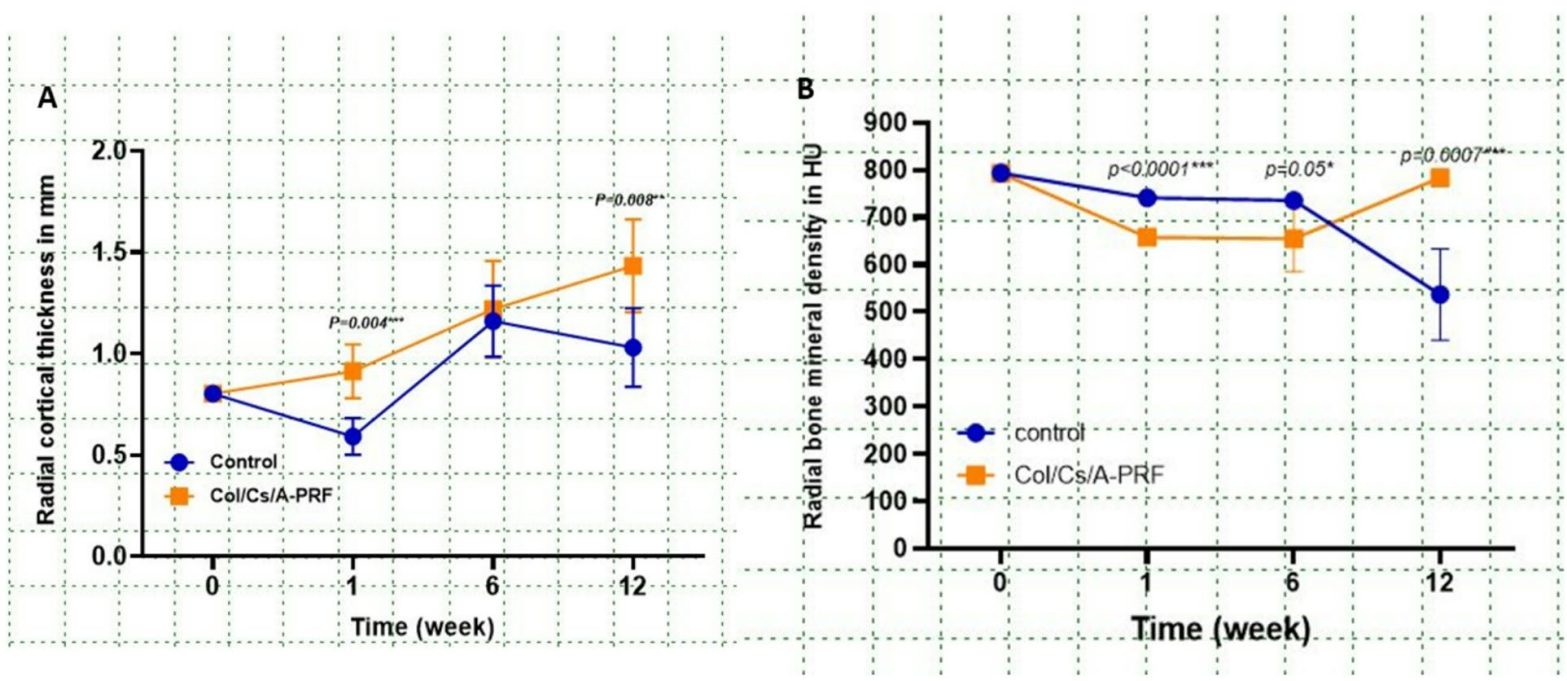
statistical graphs illustrate (**A**) Radial cortical thickness and (**B**) radial bone mineral density (HU) measured at baseline and at 1, 6, and 12 weeks postoperatively in the control and Col/Cs/A-PRF groups. Data are presented as mean ± SD. (*n* = 8 per group).

In the C group, RCT decreased at week 1 (0.60 ± 0.15 mm) compared to baseline (0.79 ± 0.04 mm), followed by a progressive increase peaking at week 6 (1.13 ± 0.16 mm) and a slight decline at week 12 (1.04 ± 0.21 mm). In contrast, the Col/Cs/A-PRF group exhibited a significant early increase in RCT at week 1 (0.91 ± 0.13 mm; *p* < 0.001), continued thickening at week 6 (1.29 ± 0.15 mm), and at week 12 (1.4 ± 0.05 mm). (Fig. 2.**A)**

Radial bone mineral density (RBMD) demonstrates temporal variations between the C and Col/Cs/A-PRF-treated groups over 12 weeks. At baseline, both groups exhibited similar BMD values (~ 800 HU). By week 1, the Col/Cs/A-PRF group showed a significant reduction in BMD (~ 650 HU) compared to the C group (~ 750 HU), with a highly significant difference (*p* < 0.0001). At week 6, BMD levels were nearly comparable between groups (*p* = 0.05). By week 12, the Col/Cs/A-PRF group exhibited a substantial increase in BMD (~ 780 HU), while the C group experienced a marked decline (~ 550 HU), with a statistically significant difference (*p* = 0.0007). **(**Fig. 2.**B)**

**Fig. 3:**
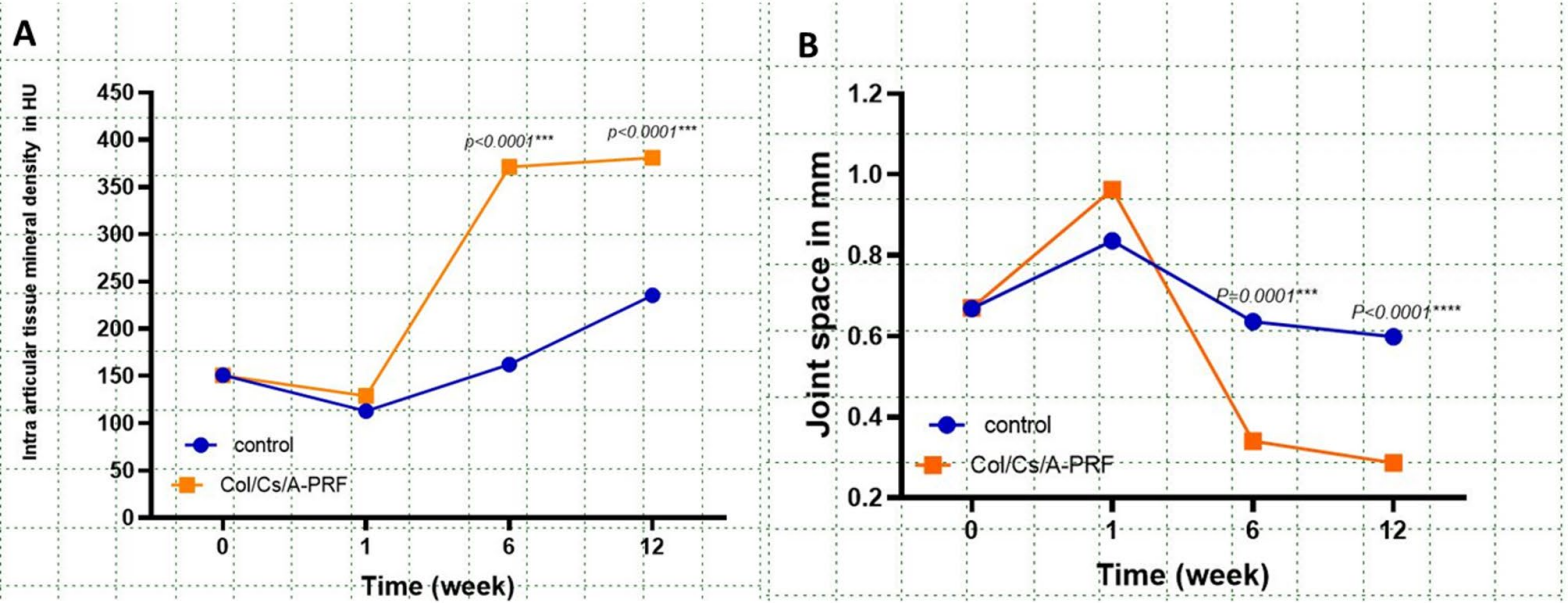
A statistical graph shows intra-articular changes following antebrachiocarpal arthrodesis. (**A**) Intra-articular tissue mineral density (HU) and (**B**) joint space width (mm) measured at baseline and at 1, 6, and 12 weeks postoperatively in the control and Col/Cs/A-PRF groups. Data are presented as mean ± SD. (*n* = 8 per group).

The progression of intra-articular tissue mineral density (MD). At baseline, both groups show similar MD values, around 150 HU. Over the first week, both groups experienced slight decreases in MD, remaining close to baseline levels, with no notable divergence or statistical difference observed. However, by week 6, a significant distinction emerges: while the C group maintains a relatively stable MD, the Col/Cs/A-PRF group shows a sharp increase, reaching approximately 380 HU, a dramatic rise that is statistically highly significant (*p* < 0.0001). By week 12, the C group’s MD increases slightly to about 220 HU, whereas the Col/Cs/A-PRF group’s MD remains elevated. **(**Fig. 3.**A)**

Regarding the joint space narrowing in both groups. At week 0, both groups started with comparable joint space values (~ 0.68 mm). By week 1, the Col/Cs/A-PRF group showed a transient increase in joint space (~ 1.0 mm), slightly exceeding that of the C group (~ 0.85 mm), However, a marked reduction in joint space was observed in the treated group by week 6 (~ 0.35 mm), significantly lower than the C group (~ 0.63 mm), with a highly significant difference (*p* < 0.0001). This narrowing continued at week 12 (~ 0.3 mm in the treated group vs. ~0.6 mm in the control), with the difference remaining statistically significant (*p* < 0.0001). **(**Fig. 3.**B)**

**Fig. 4:**
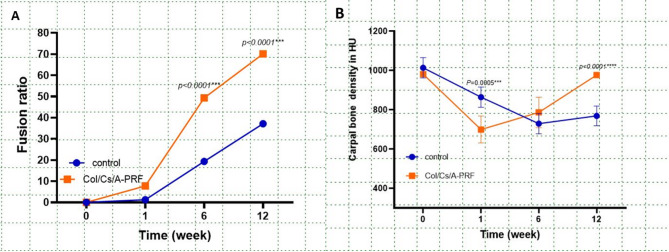
Statistical graph demonstrates (**A**) Fusion ratio (%) and (**B**) carpal bone mineral density (HU) measured at baseline and at 1, 6, and 12 weeks postoperatively in the control and Col/Cs/A-PRF groups. Data are presented as mean ± SD (*n* = 8 per group).

**Fig. 5:**
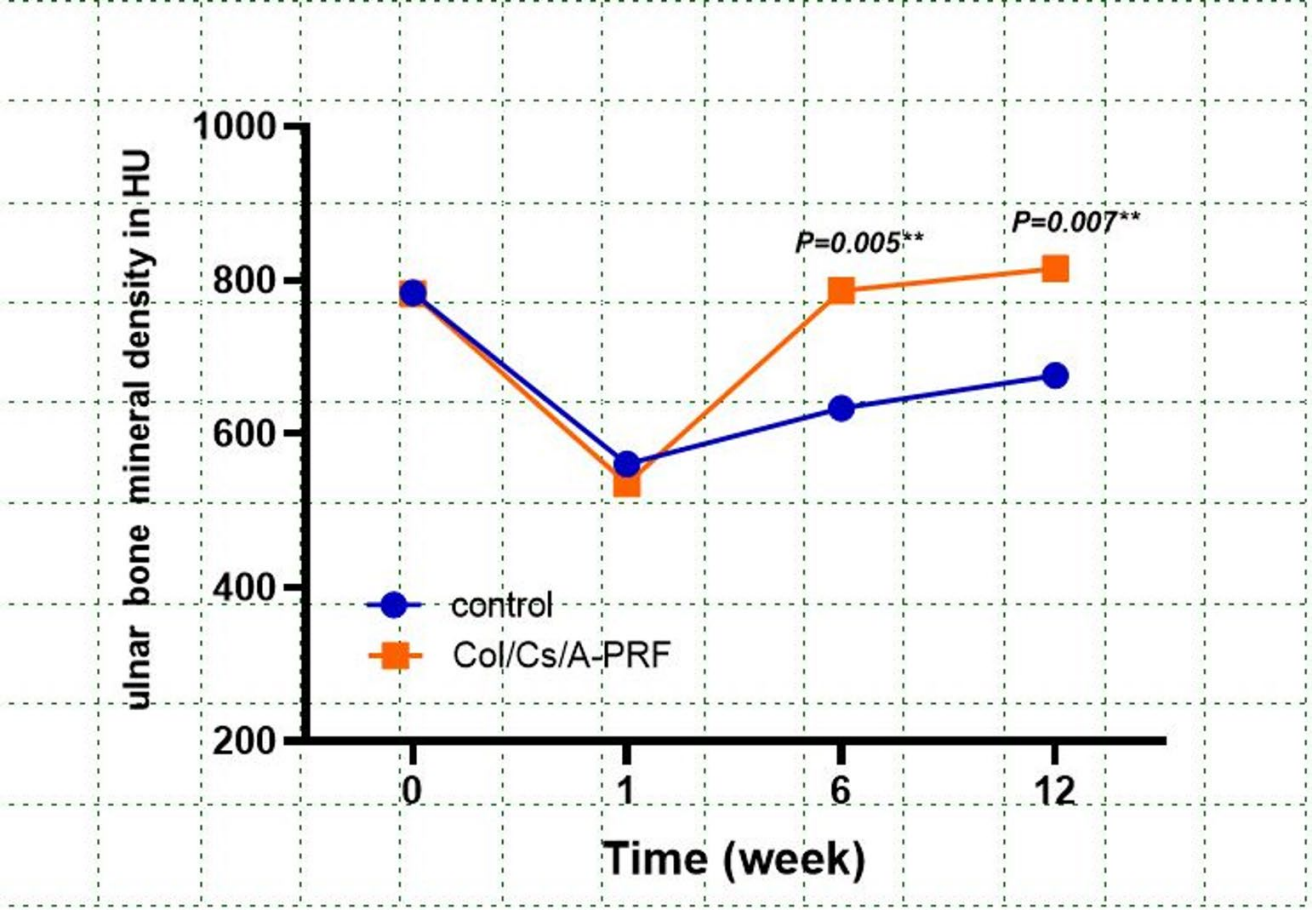
Statistical graph demonstrates Ulnar bone mineral density (HU) measured at baseline and at 1, 6, and 12 weeks postoperatively in the control and Col/Cs/A-PRF groups. Data are presented as mean ± SD (*n* = 8 per group).

**Fig. 6:**
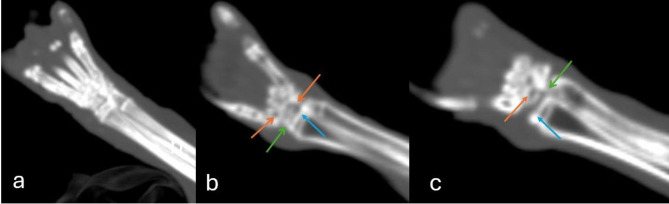
Coronal oblique reformatted CT images of the radio-carpal joint after intervention (**a**) after one week, (**b**) after 1.5 months, and (**c**) 3 months intervals showing cystic erosions at carpal bones (orange arrows), progressive narrowing of the joint space (green arrows), increased bone density (blue arrows) and fusion ratio about 50% and 70.2% at 1.5- and 3-months intervals respectively.

**Fig. 7:**
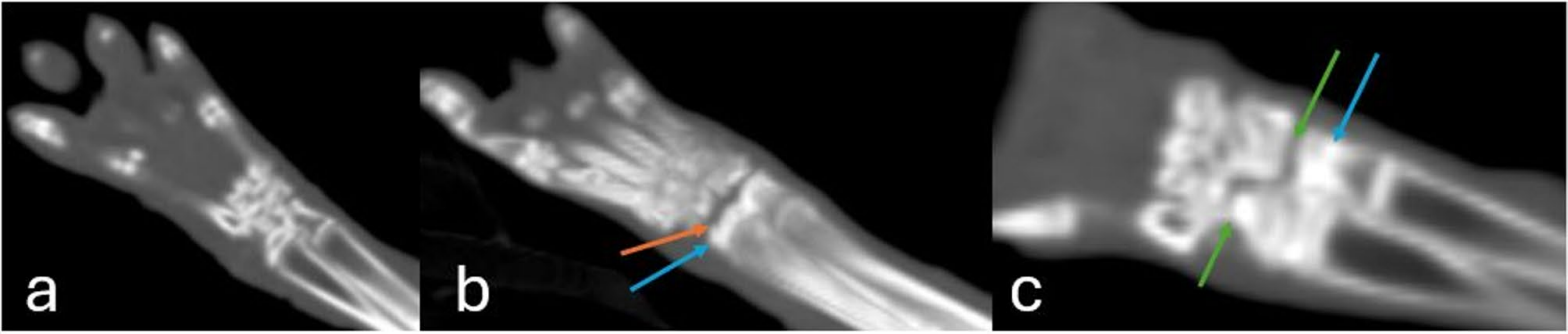
Coronal oblique reformatted CT images of the radio-carpal joint after intervention (**a**) after one week, (**b**) after 1.5 months, and (**c**) 3 months intervals showing still open joint space at 1.5 months exam (b, orange arrow) and delayed progressive narrowing of the joint space (green arrows), increased bone density (blue arrows), incomplete osseous bridging and fusion ratio about 69.5% at 3-months exam.

**Fig. 8:**
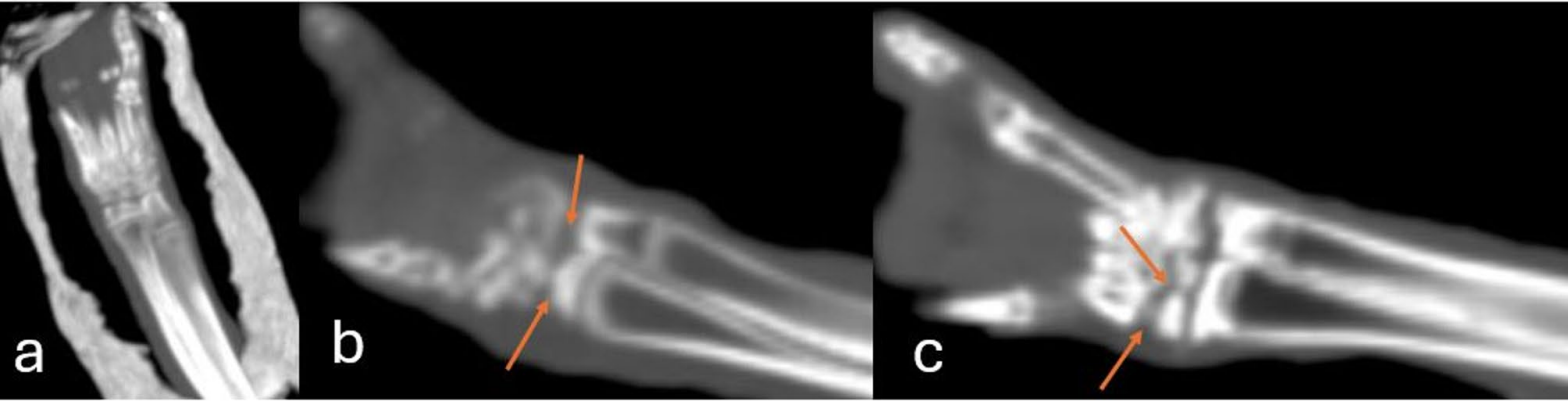
Coronal oblique reformatted CT images of the radio-carpal joint after intervention (**a**) after one week, (**b**) after 1.5 months, and (**c**) 3 months intervals showing still open joint space (orange arrows), no osseous bridging (orange arrows) and fusion ratio about 34% and 28% at 1.5- and 3-months intervals, respectively.

At baseline, both groups exhibited negligible fusion ratios. By week 1, the Col/Cs/A-PRF group demonstrated a statistically significant increase in the fusion ratio compared to the C group (*p* < 0.0001). This trend continued over time, with the Col/Cs/A-PRF group showing markedly higher fusion ratios at both week 6 and week 12 (*p* < 0.0001 for both time points. At week 12, the treated group achieved a mean fusion ratio approaching 70% (Fig. 6), Fig. 7), significantly outperforming the C group, which reached approximately 35% (Fig.4.A) (Fig. 5) (Fig. 8).

For carpal bone density, at baseline, both groups began with similar density values (~ 1000 HU). By week 1, a significant reduction was noted in the Col/Cs/A-PRF group (~ 700 HU) compared to the C group (~ 860 HU), with a statistically significant difference (*p* = 0.0005), At week 6, bone density in the treated group showed improvement (~ 790 HU), slightly exceeding that of the control (~ 750 HU). By week 12, the Col/Cs/A-PRF group exhibited a substantial increase in carpal bone density (~ 990 HU), significantly higher than the control (~ 780 HU), with a highly significant difference (*p* < 0.001). **(**Fig. 4.**B)**

The changes in ulnar bone mineral density (UBMD) at baseline, both groups start with similar BMD values (~ 800 HU). However, by week 1, the Col/Cs/A-PRF group shows a significant decline in BMD to approximately 550 HU, while the control group also decreases but to a lesser extent (~ 650 HU). By week 6, the Col/Cs/A-PRF group demonstrates a notable recovery, with BMD increasing to about 750 HU, surpassing the control group’s BMD (~ 600 HU) and showing a statistically significant difference (*p* = 0.005). By week 12, both groups show further stabilization, with the Col/Cs/A-PRF group maintaining a higher BMD (~ 800 HU) compared to the control group (~ 650 HU). **(**Fig. 5.**)**

## Discussion

The present study represents a controlled experimental approach of a surgically induced arthrodesis in a rabbit model to evaluate bone fusion. While this model does not replicate chronic degenerative joint disease, it allows standardized assessment of osteogenesis and fusion under reproducible conditions similar to clinical scenarios involving severe cartilage loss, joint instability, or osteochondral damage requiring arthrodesis. Collagen and chitosan are natural polymers widely recognized for their biocompatibility, biodegradability, and osteoconductivity, making them valuable scaffolds for bone regenerative strategies^25,26^..

Scaffolds incorporating natural polymers such as collagen and chitosan have been shown to effectively enhance osteogenic differentiation, mimic the bone microenvironment, supporting cell adhesion, proliferation, and differentiation into bone-forming cells, making them promising candidates for bone tissue engineering^27^.

Clinical and preclinical studies show that such tissue-engineered scaffolds can significantly improve bone regeneration and fusion rates in challenging defects, such as those seen in spinal fusion or critical-sized bone injuries and arthrodesis of the antebrachiocarpal joint^4,23,28^.

Advanced platelet-rich fibrin is a newer type of PRF that primarily differs from traditional PRF in its preparation process, resulting in a looser fibrin network and a higher, more sustained release of growth factors^29^. Compared to traditional PRF, A-PRF has been shown to enhance bone regeneration by promoting greater angiogenesis, osteoblast proliferation, and bone deposition, leading to improved healing outcomes in both animal and clinical studies^30^. A-PRF also provides a longer-lasting supply of growth factors such as PDGF, VEGF, BMP-2, and TGF-β1, which are crucial for bone healing and remodeling^31^. This prolonged release supports continuous bone regeneration and integration with graft materials^31^. Mediators such as osteocalcin, osteonectin, fibrinogen, vitronectin, fibronectin, and thrombospondin within an A-PRF, together with immune cells, provide a bioactive environment that enhances regenerative potential by supporting intercellular communication, guiding tissue-specific macrophage differentiation, and promoting bone and soft tissue healing^32^.

The time at which a centrifugation procedure begins following blood draw is critical to optimize the size outcomes of A-PRF membranes. In general, approximately 15 s is required per tube to harvest 9–10 cc of blood. Therefore, a 60- to 90-s interval between blood draw and the start of centrifugation should be a parameter that is respected by clinicians to avoid significant changes in the macroscopic morphology and size of fabricated PRF membranes^33^.

Cortical thickness is a key morphological factor that affects bone strength, as it is closely linked to the bone’s capacity to resist mechanical forces. Together with external bone measurements, such as outer diameter, it plays a major role in maintaining the structural and functional stability of the skeletal system^34^. In the current study, the Col/Cs-A-PRF group showed significantly higher radial cortical thickness and bone mineral density (BMD) at weeks 6 and 12 compared to the control group. This proposes that the scaffold not only supported early callus formation but also promoted ongoing bone remodeling and consolidation, in line with previous reports on the synergistic effects of PRF-enriched scaffolds in bone regeneration^35^.

In a previous study, the fusion ratio achieved using collagen–chitosan was approximately 65%^23^. while enrichment of COL/Cs with A-PRF resulted in a marked increase in the fusion ratio to reach about 70%.

This aligns with earlier studies demonstrating that tissue-engineered scaffolds can enhance fusion rates in challenging defects^4^. This enhancement is also attributed to the growth factor-rich nature of PRF, which supports angiogenesis, stimulates mesenchymal stem cell differentiation, and provides a scaffold for cellular migration, thus promoting more rapid and robust bone formation^36^.

Radial bone mineral density RBMD showed a biphasic pattern in the Col/Cs-A-PRF group, with an initial decrease followed by a marked increase by week 12. This early decline represents the resorption phase, while the subsequent Col/Cs-A-PRF, particularly A-PRF, enhances bone regeneration by promoting osteogenic differentiation and supporting the processes that lead to increased bone mineral density^37^. Similar trends were observed in ulnar BMD and carpal bone density.

Prior investigations have reported that the use of collagen/chitosan alone resulted in a joint space width of approximately 0.5 mm^23^, whereas the addition of A-PRF was associated with an approximately 0.2 mm increase in joint space narrowing. increased bone density, and partial to complete osseous bridging. This outcome supports the hypothesis that the incorporation of collagen and chitosan with A-PRF synergistically enhances early extracellular matrix deposition, supports mesenchymal cell recruitment, promotes endochondral ossification, and modulates the local microenvironment to favor osteogenesis and structural joint integration during arthrodesis^37,38^.

MSCT-derived radiodensity measurements strongly correlate with histomorphometric analysis, validating the accuracy of imaging-based assessments for bone quality and healing^20,39^, This correlation means that MSCT can serve as a surrogate for more invasive histological evaluations, making it especially valuable in both experimental and translational orthopedic research^39^. Moreover, the use of MSCT provides a powerful, non-invasive method for high-resolution monitoring of bone healing dynamics over time. MSCT enables quantitative assessment of bone regeneration through measurements such as HU values, cortical thickness, and fusion ratio, offering reliable insights into the progression of conditions like ankylosis or bone fusion^20,40]^. It also enables longitudinal, in vivo evaluation with repeated measurements over time, which is not feasible with histological analysis^41^.

## Conclusion

The current study indicates that the addition of a Col/Cs/A-PRF composite scaffold significantly improves bone regeneration and fusion ratio in a rabbit model of antebrachiocarpal arthrodesis. MSCT evaluations revealed better bone integration, increased cortical thickness, gradual joint space narrowing, and higher fusion rates.

## Clinical relevance and future directions

This study supports the use of bioactive scaffolds combined with growth factor-rich matrices in enhancing bone fusion outcomes. The successful application of the Col/Cs/A-PRF composite in a small animal model suggests its potential translatability to larger animals and possibly to human orthopedic applications, particularly in cases of complex joint defects or failed conservative treatments.

## Study limitations

Further studies are necessary to evaluate long-term integration, mechanical strength, and potential translational applications in larger animal models or human clinical trials. Future studies should also incorporate Weight-bearing and gait analysis assessments. Accordingly, MSCT adopted in the present study provides a valid, reproducible, and minimally invasive method for monitoring bone fusion in preclinical orthopedic research. Nevertheless, histological assessment remains essential for detailed cellular-level characterization and precise tissue differentiation, and its absence is therefore acknowledged as an important limitation of the current study.

## Data Availability

All data generated or analyzed during this study are included in this published article and its supplementary materials, including the graphic abstract presented as an attached supplementary file.

## Abbreviations

A-PRF: Advanced platelet-rich fibrin
Col/Cs: Collagen–chitosan
MSCT: Multislice Computed tomography
RCT: Radial cortical thickness
RBMD: Radial bone mineral density
UBMD: Ulnar bone mineral density
BMD: Bone mineral density
MD: Mineral density
ROI: Region of interest
HU: Hounsfield units
CT: Computed tomography
PRF: Platelet-rich fibrin
PDGF: Platelet-derived growth factor
VEGF: Vascular endothelial growth factor
TGF-β1: Transforming growth factor beta 1
IGF-1: Insulin-like growth factor 1
bFGF: Basic fibroblast growth factor
BMP-2: Bone morphogenetic protein 2
ALP: Alkaline phosphatase
C: Control group
Col/Cs/A-PRF: Collagen–chitosan + advanced platelet-rich fibrin
ARRIVE: Animal research: reporting of in vivo experiments
MUACUC: Mansoura university animal care and use committee
